# The Algal Polysaccharide Ulvan Induces Resistance in Wheat Against *Zymoseptoria tritici* Without Major Alteration of Leaf Metabolome

**DOI:** 10.3389/fpls.2021.703712

**Published:** 2021-09-06

**Authors:** Marlon C. de Borba, Aline C. Velho, Alessandra Maia-Grondard, Raymonde Baltenweck, Maryline Magnin-Robert, Béatrice Randoux, Maxime Holvoet, Jean-Louis Hilbert, Christophe Flahaut, Philippe Reignault, Philippe Hugueney, Marciel J. Stadnik, Ali Siah

**Affiliations:** ^1^Laboratory of Plant Pathology, Agricultural Science Center (UFSC-CCA), Federal University of Santa Catarina, Florianópolis, Brazil; ^2^Joint Research Unit N° 1158 BioEcoAgro, ULCO, INRAE, University of Lille, Université Liège, UPJV, University of Artois, Lille, France; ^3^Unité de Chimie Environnementale et Interactions sur le Vivant (EA 4492), Université du Littoral Côte d’Opale, Calais, France; ^4^INRAE, SVQV UMR-A1131, Université de Strasbourg, Colmar, France

**Keywords:** septoria tritici blotch, green seaweed, induced resistance, MALDI-TOF-MS, gene expression, UHPLC-MS, metabolomics

## Abstract

This study aimed to examine the ability of ulvan, a water-soluble polysaccharide from the green seaweed *Ulva fasciata*, to provide protection and induce resistance in wheat against the hemibiotrophic fungus *Zymoseptoria tritici*. Matrix-assisted laser desorption/ionization-time-of-flight-mass spectrometry (MALDI-TOF-MS) analysis indicated that ulvan is mainly composed of unsaturated monosaccharides (rhamnose, rhamnose-3-sulfate, and xylose) and numerous uronic acid residues. In the greenhouse, foliar application of ulvan at 10 mg.ml^–1^ 2 days before fungal inoculation reduced disease severity and pycnidium density by 45 and 50%, respectively. Ulvan did not exhibit any direct antifungal activity toward *Z. tritici*, neither *in vitro* nor *in planta*. However, ulvan treatment significantly reduced substomatal colonization and pycnidium formation within the mesophyll of treated leaves. Molecular assays revealed that ulvan spraying elicits, but does not prime, the expression of genes involved in several wheat defense pathways, including pathogenesis-related proteins (β-1,3-endoglucanase and chitinase), reactive oxygen species metabolism (oxalate oxidase), and the octadecanoid pathway (lipoxygenase and allene oxide synthase), while no upregulation was recorded for gene markers of the phenylpropanoid pathway (phenylalanine ammonia-lyase and chalcone synthase). Interestingly, the quantification of 83 metabolites from major chemical families using ultra-high-performance liquid chromatography-mass spectrometry (UHPLC-MS) in both non-infectious and infectious conditions showed no substantial changes in wheat metabolome upon ulvan treatment, suggesting a low metabolic cost associated with ulvan-induced resistance. Our findings provide evidence that ulvan confers protection and triggers defense mechanisms in wheat against *Z. tritici* without major modification of the plant physiology.

## Introduction

The Septoria tritici blotch, caused by the hemibiotrophic fungus *Zymoseptoria tritici*, is one of the most devastating diseases of wheat (*Triticum aestivum* L.) worldwide (Fones and Gurr, 2015). The reduction in the photosynthetic leaf area caused by the disease can lead to yield losses of up to 50% or 5–10% when integrated disease management is implemented (Fones and Gurr, 2015). Currently, disease control mainly relies on the use of conventional fungicides (Torriani et al., 2015) and partially resistant cultivars (Ors et al., 2018). However, due to the high genetic diversity and adaptability of *Z. tritici*, the fungus frequently develops resistance to fungicides and often overcomes host resistance, making it one of the most difficult plant pathogens to control (Fones and Gurr, 2015; Torriani et al., 2015). Hence, looking for complementary alternatives, such as the use of plant resistance inducers from natural origin, has been recognized as an eco-friendly strategy to control the STB disease, thus contributing to safer food production.

The life cycle of *Z. tritici* is characterized by two distinct stages, namely, biotrophic (or symptomless latent phase) and necrotrophic phases (Steinberg, 2015). Leaf penetration occurs most frequently by hyphae emerging from germinating and surface-attached conidia that enter through stomata (Steinberg, 2015) or directly *via* the anticlinal and periclinal epidermal cells (Siah et al., 2010a). During this asymptomatic period, the fungus grows extremely slowly between the mesophyll cell layers of the leaf (Keon et al., 2007; Rudd et al., 2015). The transition from the biotrophic to the necrotrophic phase takes place suddenly around 10–15 days after infection and coincides with a sharp increase in fungal growth rate and biomass (Keon et al., 2007; Siah et al., 2010a). This may be supported by the increased apoplastic nutrient availability as a consequence of the loss of host cell-wall integrity (Keon et al., 2007). Finally, the fungus produces asexual spores in the dark-brown pycnidia formed under colonized substomatal cavities (Siah et al., 2010a; Steinberg, 2015).

Ulvan is a water-soluble sulfated heteropolysaccharide extracted from the cell walls of the green macroalgae *Ulva* spp. and is composed mainly of rhamnose, xylose, glucose, uronic acid, and sulfate (Paulert et al., 2009; de Freitas et al., 2015). Ulvan is known to exhibit a broad spectrum of biological activities, including stimulation of plant growth and defense responses (Stadnik and de Freitas, 2014). For instance, when applied onto bean leaves, ulvan is able to reduce the severity of anthracnose (Paulert et al., 2009; de Freitas and Stadnik, 2012), rust (Delgado et al., 2013), and powdery mildew (Jaulneau et al., 2011). In addition, it has shown promising results in the resistance of wheat and barley against the powdery mildew caused by *Blumeria graminis* (Paulert et al., 2010). However, the potential of green algal polysaccharides for controlling STB in wheat has never been examined.

The mode of action of the resistance inducers differs from that of traditional pesticides because they do not directly target the pathogen through antifungal activity, but they inhibit its development indirectly *via* the induction of natural plant defense mechanisms. The status of induced resistance in plants could be related to a direct elicitation of defense responses (i.e., elicitor effect), which occurs in the absence of infection, or to a plant sensitized by a priming agent (i.e., priming effect). This, in turn, results in the expression of earlier and/or stronger basal defense mechanisms upon pathogen attacks (Walters et al., 2005). Although not well understood yet, ulvan seems to induce resistance through the elicitation of defense responses in dicot plants (Cluzet et al., 2004; de Freitas and Stadnik, 2012) and priming in monocot plants (Paulert et al., 2010).

Wheat defense mechanisms against *Z. tritici* are complexes. It has been suggested that the upregulation of pathogenesis-related (PR) proteins (β-1,3-glucanase, *PR-2*; chitinase, *PR-3*) (Adhikari et al., 2007; Shetty et al., 2009) and reactive oxygen species (ROS) metabolism (oxalate oxidase, *OXO*) (Shetty et al., 2007) plays a major role in incompatible interactions, while induction of phenylpropanoids (phenylalanine ammonia-lyase, *PAL*; chalcone synthase, *CHS*) is sometimes observed in partially resistant wheat cultivars (Ors et al., 2018). On the other hand, the expression of genes involved in the octadecanoid pathway (lipoxygenase, *LOX*; allene oxide synthase, *AOS*) seem to be downregulated soon after fungal infection and further upregulated (Rudd et al., 2015; Somai-Jemmali et al., 2017). Although not well explained, the expression of these defense-related genes has been associated with the induction of wheat resistance against *Z. tritici* after the application of resistance inducers (Ors et al., 2019; Somai-Jemmali et al., 2020).

Metabolomics is an emerging and powerful tool aiming at the comprehensive analysis of low-molecular-weight metabolites. Metabolomics has been used for an overview of plant status after several biotic and abiotic stresses, particularly to decipher host resistance or susceptibility following pathogen infection (Rudd et al., 2015; Seybold et al., 2020). Knowing metabolic profiles during plant resistance induction might allow a better characterization of activated defense mechanisms. To date, little is known about the wheat-induced resistance against *Z. tritici* using natural inducers. Thus, the objective of this study was to evaluate the potential of ulvan to protect wheat against *Z. tritici* and determine its effect on plant gene expression and metabolic profile during the biotrophic phase of the fungus life cycle.

## Materials and Methods

### Biological Materials

Wheat plants (*Triticum aestivum* L.) of cv. Alixan, which are susceptible to *Z. tritici* (Ors et al., 2018), were purchased from Limagrain (Saint-Beauzire, France) and used in all experiments. The aggressive monosporic strain of *Z. tritici* T02596 (Mejri et al., 2018) was used in all wheat infection bioassays. The fungus was grown on a potato dextrose agar (PDA) medium at 18°C at a 12-h photoperiod for 7 days. Then, Petri dishes were flooded with 10 ml of distilled water, and the spore suspension was filtered two times to remove mycelial fragments. Spore concentration was determined using a Malassez counting chamber (Paul Marienfeld, Lauda-Königshofen, Germany) and adjusted as needed. Ulvan was obtained as previously described by Paulert et al. (2009) from the green seaweed *Ulva fasciata* and collected on August 2017 at the Barra da Lagoa beach in Florianópolis-SC, Brazil. Briefly, 100 g of dried algae was autoclaved for 2 h at 110°C in 1 L of distilled water. The resulting aqueous solution was filtered and the polysaccharide was precipitated two times with ethanol (3 v) at −20°C: first for 24 h, and afterward for 48 h. The second precipitate of ulvan was collected, dried, and kept at 5°C until use.

### Matrix-Assisted Laser Desorption/Ionization-Time-of-Flight-Mass Spectrometry (MALDI-TOF-MS)

Matrix-assisted laser desorption/ionization-time-of-flight-mass spectrometry (MALDI-TOF-MS) analysis was performed using the AutoflexSpeed^TM^ (Bruker, Berlin, Germany) mass spectrometer running the Flexcontrol 3.4 software (Bruker). The mass spectrometer was calibrated according to the recommendations of the manufacturer. Mass spectra were acquired in the negative linear-ion mode using the automatic negative linear method of the manufacturer across a mass-to-charge (*m*/*z*) ratio of 100–2,000 atomic mass unit. The mass spectra correspond to mass signals summed from 2,000 laser shots in 100 shot steps performed randomly on different areas of the spot. Ulvan was solubilized at 10 mg.ml^–1^ in ultrapure (MQ) water, while 2,5-dihydroxybenzoic acid (DHB), used as the MALDI matrix, was dissolved at 10 mg.ml^–1^ in acetonitrile/water (3:7; v/v). One microliter of ulvan solution and 1 μl of DHB matrix solution were spotted together on a 384-well polished steel MALDI plate. Samples were allowed to dry and co-crystallize at room temperature before they were loaded into the MALDI-TOF mass spectrometer.

### *In vitro* Antifungal Activity of Ulvan on *Z. tritici*

The direct antifungal effect of ulvan on *Z. tritici* was assessed on both spore germination and mycelial growth. For the spore germination assay, an aliquot of 200 μl of 1 × 10^4^ spore ml^–1^ of *Z. tritici* was spread on PDA plates amended or not with ulvan at 10 mg.ml^–1^. After a 1-day incubation period at 18°C in the dark, the percentage of spore germination was randomly determined from 100 spores using a light microscope at 400× magnification (Siah et al., 2010b). Regarding mycelial growth assay, an aliquot of 5 μl of 1 × 10^5^ spore ml^–1^ of the fungus was deposited on the middle of Petri dishes containing PDA amended or not with ulvan at 10 mg.ml^–1^. After an incubation period of 10 days at 18°C in the dark, the fungal colony diameter was scored from two perpendicular measurements using a caliper (Siah et al., 2010b).

### Plant Growth, Treatment, and Inoculation

The wheat seeds of cv. Alixan were pre-germinated in plastic boxes on moist filter paper in the dark under changing temperature conditions, according to Siah et al. (2010a). Twelve wheat germlings were transplanted to 3-L pots (15-cm diameter) containing organic compost as the substrate (Gamm Vert, France). Three pots of 12 plants (i.e., 36 plants) were used as replicates for each condition. Plants were grown under greenhouse conditions (18 ± 3°C, 16 h of light, and a photon flux density of 240 μmol.m^–2^.s^–1^) and irrigated according to their water requirements. Three-week-old plants (the third leaf fully expanded stage) were sprayed one time with distilled water (control) or with ulvan solution (10 mg.ml^–1^), both amended with 0.05% (v/v) polyoxyethylene-sorbitan monolaurate (Tween 20, Sigma-Aldrich, Saint Louis, MO, United States). A solution volume of 30 ml was delivered per pot. Two days after treatment (dat), plants were inoculated by spraying them with a 1 × 10^6^ spore ml^–1^ suspension of *Z. tritici* prepared in distilled water with 0.05% Tween 20 and kept under highly humid conditions (∼99% humidity) for 3 days (Siah et al., 2010a). A spore suspension volume of 30 ml was delivered per pot. Mock-inoculated plants were used as control. Disease severity was assessed at 21 days after inoculation (dai) by measuring the percentage of the third leaf area with STB symptoms (chlorosis and necrosis). Pycnidium density was scored on a scale from 0 to 5; with 0 = absence of pycnidia, 1 = 1–19%, 2 = 20–39%, 3 = 40–59%, 4 = 60–79%, and 5 = 80–100% of necrotic leaf area bearing pycnidia, respectively.

### *In planta* Cytological Assays

To assess fungal spore germination and epiphytic hyphal growth (fungal development on the leaf surface), 4-cm third-leaf segments were collected at 1 and 5 dai, respectively, and then immediately immersed in a solution of 0.1% Calcofluor (Fluorescence Brightener 28, Sigma-Aldrich, Saint-Quentin-Fallavier, France) and 0.1 M of Tris-HCl buffer at pH 8.5 for 5 min. Then, leaf segments were washed for 2 min in distilled water, superficially dried at room temperature, placed on a glass slide, covered with a coper slip, and observed microscopically (Eclipse 80i, Nikon, Champigny Sur Marne, France) at 400× magnification under ultraviolet illumination (emission: 365 nm; excitation: 440 nm). The percentage of germinated spores and epiphytic hyphal growth were calculated from 100 spores, which were chosen randomly on the leaf surface. Four categories of germlings were recorded: non-germinated spore (NGS), germinated spore with a short germ tube (GS-SGT), germinated spore with a well-developed germ tube (GS-DGT), and germinated spore with branched hyphae (GS-BH). Pictures were taken with a digital camera (DXM1200C, Nikon) using image capture software (Nis-Elements BR, Nikon).

To measure the colonization of the substomatal cavities, 4-cm third-leaf segments were collected at 21 dai and then bleached in a mixture of absolute ethanol and acetic acid (3:1; v/v) overnight. The cleared leaves were rehydrated in distilled water for 4 h and then fixed in lactoglycerol (lactic acid: glycerol: water; 1:1:1; v/v/v) for 20 min. The fungal structures were stained by immersing the leaf segments in 0.1% Trypan blue (Sigma-Aldrich, Saint Quentin Fallavier, France) dissolved in lactophenol-ethanol (1:2; v/v) at 50°C for 20 min. After washing, the leaf segments were fixed in lactoglycerol, placed on a glass slide, and then observed microscopically at 400× magnification. The colonization of the substomatal cavities was determined from 150 substomatal cavities chosen randomly by assessing the following cytological events: non-colonized stomata (NCS), colonized stomata but not yet filled with a pycnidium (CS), and colonized stomata filled with a pycnidium (P). Pictures were taken using the digital camera, and image capture software is mentioned above.

### RNA and Metabolite Extraction

For both plant RNA and metabolite extractions, medial third-leaf segments of approximately 200 mg were sampled at 2 and 7 dat from non-inoculated plants and at 7 dat (corresponding to 5 dai) from inoculated plants and then immediately snap-frozen in liquid nitrogen. Samples collected for RNA extraction were stored at −80°C until analysis, and those harvested for metabolite extraction were lyophilized and weighed.

Total RNA was extracted from 100 mg of frozen leaf samples using the RNeasy Plant Mini Kit (Qiagen, Venlo, Netherlands). Genomic DNA contaminating was removed using DNase RNase-Free Set (Qiagen). The RNA obtained was suspended in 60 μL of RNase-free water and quantified by measuring the absorbance at 260 nm (BioPhotometer, Eppendorf AG, Hamburg, Germany).

For metabolomic analysis, lyophilized leaf samples (i.e., 25–30 mg) were homogenized in a bead mill (TissueLyser II, Qiagen). Polar metabolites were extracted with 25 μl of methanol per mg of dry weight containing 1 μg.ml^–1^ of apigenin and 5 μg.ml^–1^ of chloramphenicol (Sigma-Aldrich) as internal standards. After vortexing two times for 30 s, the extract was sonicated for 10 min in an ultrasound bath, and then centrifugated at 12,000 × *g* at 21°C for 15 min. Finally, 150 μl of the supernatant was filtered and transferred to a vial and analyzed by ultra-high-performance liquid chromatography-mass spectrometry (UHPLC-MS).

### Reverse Transcription-Quantitative PCR (RT-qPCR) Analysis

Reverse transcription of total RNA was carried out using the High Capacity cDNA Reverse Transcription Kit (Applied Biosystems, Waltham, MA, United States) according to the protocol provided by the manufacturer. The PCR reactions were performed with the obtained cDNA to amplify seven target genes: phenylalanine ammonia-lyase (*PAL*), chalcone synthase (*CHS*), lipoxygenase (*LOX*), allene oxide synthase (*AOS*), β-1,3-endoglucanase (*PR-2*), chitinase 2 (*PR-3*), and oxalate oxidase (*OXO*) (Tayeh et al., 2015; Ors et al., 2018) (Supplementary Table 1). The β-tubulin 4 (*TUB*) (Tayeh et al., 2013) and class A Apetala 2 (*PetA*) encoding genes were used as housekeeping genes after preliminary assays. Primer efficiencies were calculated by performing real-time PCR on several dilutions of the cDNA samples (>90% of efficiency). Reactions were performed in the real-time PCR detector C1000T (Bio-Rad, Marnes-la-Coquette, France) using the following thermal profile: a denaturation cycle for 3 min at 95°C, followed by an amplification and quantification cycle repeated 39 times (10 s at 95°C for annealing, 30 s at 60°C for extension). Melting curve assays were performed from 65 to 95°C with 0.5°C.s^–1^, and melting peaks were visualized to check the specificity of each amplification. The qPCR reaction was performed in duplicates (two technical replicates) for each sample.

### UHPLC-MS Analysis

Metabolomic analyses were performed using a Dionex Ultimate 3000 UHPLC system (Thermo Fisher Scientific, Waltham, MA, United States). The chromatographic separations were performed on a Nucleodur C18 HTec column (150 mm × 2 mm, 1.8-μm particle size; Macherey-Nagel, Düren, Germany) maintained at 30°C. The mobile phase consisted of acetonitrile/formic acid (0.1%, v/v, eluant A) and water/formic acid (0.1%, v/v, eluant B) at a flow rate of 0.3 ml min^–1^. The gradient elution was programmed as follows: 0–1 min, 95% B; 1–2 min, 95–85% B; 2–7 min, 85–0% B; 7–9 min, 100% A. The sample volume injected was 1 μl. The UHPLC system was coupled to an Exactive Orbitrap mass spectrometer (Thermo Fisher Scientific), equipped with an electrospray ionization (ESI) source operating in positive mode. Parameters were set at 300°C for the ion transfer capillary temperature and 2,500 V for the needle voltages. Nebulization with nitrogen sheath gas and auxiliary gas was maintained at 60 and 15 arbitrary units, respectively. The spectra were acquired within the mass-to-charge ratio (*m*/*z*) ranging from 100 to 1,000 atomic mass unit, using a resolution of 50,000 at *m*/*z* 200 atomic mass unit. The system was calibrated internally using dibutyl-phthalate as the lock mass at *m*/*z* 279.1591, giving a mass accuracy lower than 1 ppm. The instruments were controlled using the Xcalibur software (Thermo Fisher Scientific).

Metabolites were sought based on the calculated *m/z* of the corresponding pseudo-molecular ion [M + H]^+^ from a list of metabolites of interest using a suspect screening approach (Krauss et al., 2010; Flamini et al., 2013). The previous wheat metabolome characterizations were used for a detailed analysis of the metabolites of interest in specific chemical families such as benzoxazinoids (de Bruijn et al., 2016), flavonoids (Wojakowska et al., 2013), and hydroxycinnamic acid amides (Li et al., 2018). Putative metabolite identifications were proposed based on expertized analysis of the corresponding mass spectra and comparison with published literature. Further information was retrieved from the Kyoto Encyclopedia of Genes and Genomes (KEGG^1^) and PubChem^2^ databases. Relative quantification of the selected metabolites was performed using the Xcalibur software. For some metabolites, identity was confirmed with the corresponding standard provided by Sigma-Aldrich (France). Liquid chromatography-mass spectrometry (LC-MS) grade methanol and acetonitrile were purchased from Roth Sochiel (France); water was provided by a Millipore water purification system. Apigenin and chloramphenicol were used as internal standards.

### Experimental Design and Statistical Analyses

The *in vitro* microscopic assays were conducted in a completely randomized design with five replications, each composed of one Petri dish. All experiments performed *in planta* in the greenhouse, including protection efficacy, cytological, gene expression, and metabolomic analyses, were carried out in a factorial completely randomized design with two factors: treatment (water or ulvan) and inoculation (*Z. tritici* or mock-inoculated plants). Three replications (pots), each composed of three plants, were used for each condition.

After verification of the variance homogeneity of the data sets, data were subjected to ANOVA. Comparisons between data obtained for disease severity, pycnidium density, and all assessed *in planta* cytological events, as well as for *in vitro* spore germination and mycelial growth assays, were carried out with Student’s *t*-test at a significance level of *P* ≤ 0.05. For gene expression analysis, the relative levels of each gene expression normalized to the housekeeping genes *TUB* and *PetA* were represented, at each time point, as Log10 relative expression compared to water-treated control plants (expression value in the control fixed at 1) in both non-infectious and infectious contexts. To assess the effect of the fungus alone on gene expression, water-treated non-inoculated plants were compared with water-treated inoculated ones. Comparisons between data obtained in non-infectious and infectious contexts were performed with the Wilcoxon–Mann–Whitney and Kruskal–Wallis tests at *P* ≤ 0.05, respectively. Regarding metabolomic analysis, pairwise comparisons were performed using Tukey’s honest significant difference method followed by a false discovery rate (FDR) correction, with FDR < 0.05. Heatmaps were performed using the package *ComplexHeatmap* (Gu et al., 2016) after Log2 transformation data. Principal component analysis (PCA) was constructed in the package *vegan* (Oksanen et al., 2019). The statistical analyses were performed using the software R version 3.5.1 (R Core Team, 2018). All experiments were repeated three times and the values presented in this study are the average of the three experiments.

## Results

### The Algal Polysaccharide Ulvan Mainly Encompasses Unsaturated Monosaccharides and Numerous Uronic Acid Residues

**FIGURE 1:**
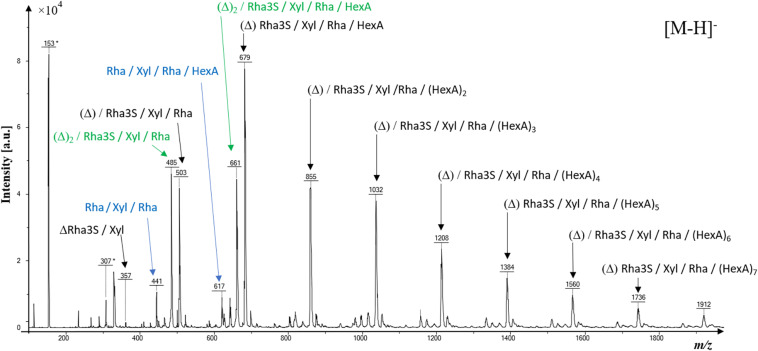
Negative linear matrix-assisted laser desorption/ionization-time-of-flight-mass spectrometry (MALDI-TOF-MS) of ulvan (second precipitate). All mentioned *m/z* ratios correspond to [M-H] ^–^ ions. Note that the *m/z* * correspond to *m/z* of the 2,5-dihydroxybenzoic acid (DHB) matrix negative ions. Rhamnose (Rha); Rhamnose-3-sulfate (Rha3S); Xylose (Xyl); Hexuronic acid (HexA); Unsaturation of monosaccharides are depicted as (Δ).

The molecular heterogeneity of ulvan oligosaccharides (*m*/*z* < to 2,000 atomic mass unit) was assessed by a linear negative MALDI-TOF-MS analysis (Figure 1 and Supplementary Table 2). The most intense [M-H]^–^ mass signals were assigned to three series (in black, blue, and green) of ulvan monosaccharide composition. The main series (in black) of ulvan oligosaccharides is composed of one unsaturation (Δ, where unsaturation can be located either on Rha3S, Rha, or HexA), one Rha3S, one Xyl, one Rha, and several hexuronic acid residues ranging from one to at least eight. Two oligosaccharide substructures (in green) were composed of two unsaturation (Δ)_2_, Rha3S, Xyl, Rha, and (HexA)_0–1_ residues, and two other minor substructures were composed of Rha, Xyl, Rha, and (HexA)_0–1_ residues (in blue) (Figure 1).

### Ulvan Protects Wheat Against *Z. tritici* and Reduces Both Host Colonization and Fungal Sporulation

**FIGURE 2:**
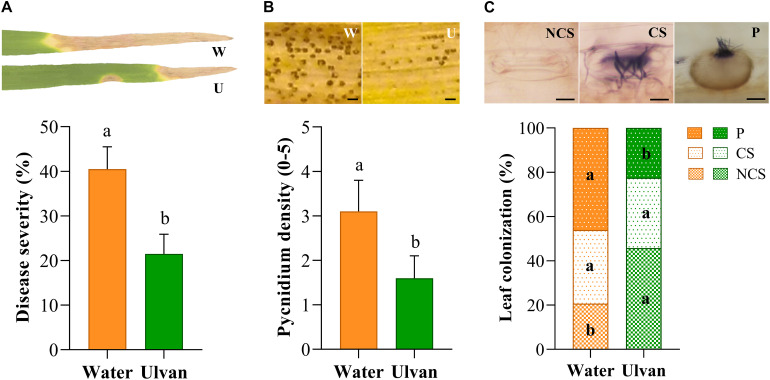
Septoria tritici blotch severity **(A)**, pycnidium density **(B)**, and colonization of substomatal cavities **(C)** on the third leaves of the wheat (cv. Alixan) plants pre-sprayed with water (control) or ulvan (10 mg.ml^–1^) and evaluated at 21 days after inoculation with *Zymoseptoria tritici.* Plants were inoculated 2 days after treatment. Letters indicate significant differences according to Student’s *t*-test at *P* ≤ 0.05. Bars represent the standard deviations of the mean. W, water; U, Ulvan; NCS, non-colonized stomata; CS, colonized stomata but not yet filled with a pycnidium; P, colonized stomata filled with a pycnidium. Scale bar = 100 μm **(B)** and 10 μm **(C)**.

The protection efficacy of ulvan in wheat against *Z. tritici* was evaluated in the greenhouse by spraying wheat plants of cv. Alixan with the fungus 2 days before inoculation. The first STB disease symptoms (chlorotic blotches and necrotic spots) appeared at 14 and 16 dai on the water- and ulvan-treated leaves, respectively. At 21 dai, the percentage of necrotic leaf area and index of pycnidium coverage reached 41% and 3.1 in control plants, respectively. Ulvan spraying significantly reduced disease severity by 45% (Figure 2A) and pycnidium density by 50% (Figure 2B) when compared to the control. Histopathological staining using Trypan blue at 21 dai revealed that the rates of non-colonized substomatal cavities were 55% higher in ulvan-treated leaves in comparison with water-treated ones (Figures 2C, 4E,F). Likewise, plants treated with ulvan showed a significant reduction in 51% in pycnidium formation when compared with the control (Figures 2C, 4G,H).

### Ulvan Did Not Display Any Direct Antifungal Activity Toward *Z. tritici* Spores and Mycelium

**FIGURE 3:**
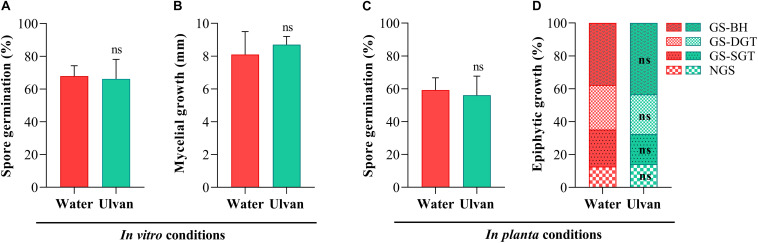
Rates of spore germination **(A)** and mycelial growth **(B)** of *Zymoseptoria tritici* assessed *in vitro* at 1 and 10 days of incubation, respectively, on potato dextrose agar medium amended with water (control) or ulvan (10 mg.ml^–1^), and spore germination **(C)** and epiphytic hyphal growth **(D)** of *Z. tritici* examined *in planta* at 1 and 5 days after inoculation, respectively, on the third leaves of the wheat plants (cv. Alixan) pre-treated with water (control) or ulvan (10 mg.ml^–1^) 2 days before fungal inoculation. ns, not significant when compared to the control according to Student’s *t*-test at *P* ≤ 0.05. NGS, non-germinated spore; GS-SGT, germinated spore with a short germ tube; GS-DGT, germinated spore with a well-developed germ tube; GS-BH, germinated spore with branched hyphae.

The direct antifungal effect of ulvan on *Z. tritici* was assessed both *in vitro* and *in planta*. *In vitro* assays highlighted that the percentage of germinated spores did not significantly differ among the PDA plates amended (66%) or not (68%) with ulvan at 10 mg.ml^–1^ (Figure 3A). A similar pattern was observed for mycelial growth since no significant effect was observed between fungal colony diameters scored in PDA plates supplemented (8.7 mm) or not (8.1 mm) with ulvan at 10 mg.ml^–1^ (Figure 3B).

**FIGURE 4:**
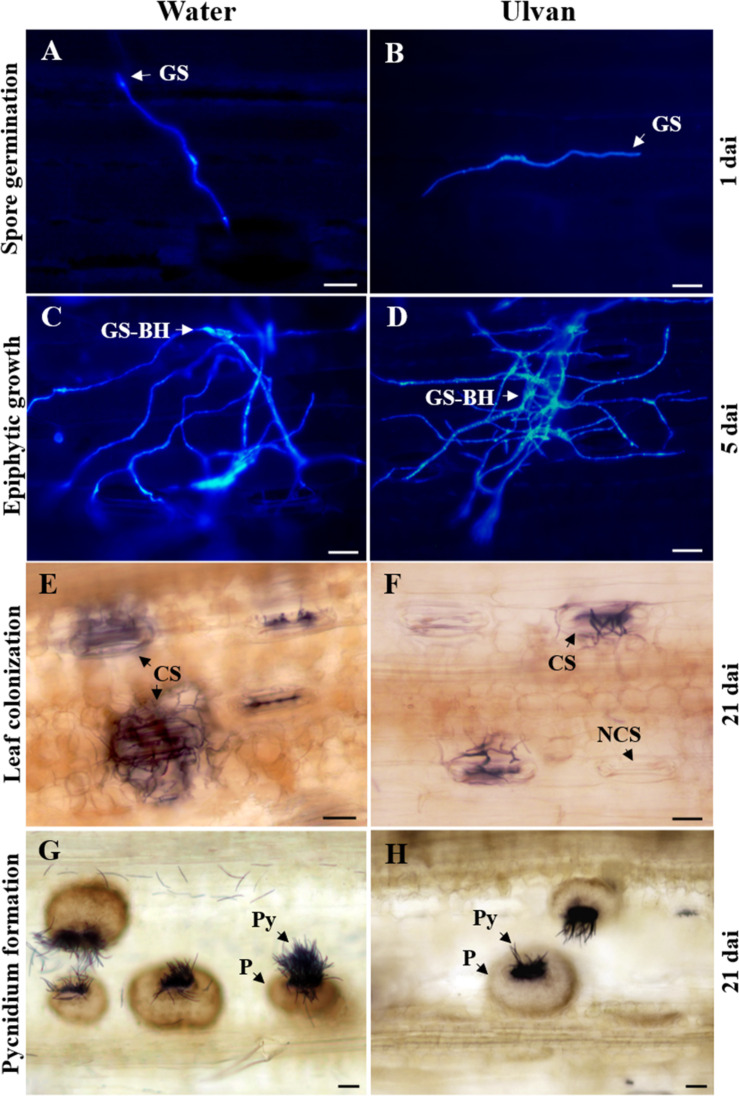
Illustration of spore germination **(A,B)**, epiphytic hyphal growth **(C,D)**, colonization of substomatal cavities **(E,F),** and pycnidium formation **(G,H)** of *Zymoseptoria tritici* on wheat leaves (cv. Alixan) pre-treated with water (control) or ulvan (10 mg.ml^–1^) and assessed at 1, 5, and 21 days after inoculation, respectively. Plants were inoculated 2 days after treatment. Fungal structures were stained with Calcofluor **(A–D)** and Trypan blue **(E–H)** and visualized under fluorescence microscopy and light microscopy, respectively. GS, germinated spore; GS-BH, germinated spore with branched hyphae; NCS, non-colonized stomata; CS, colonized stomata but not yet filled with a pycnidium; P, colonized stomata filled with a pycnidium; Py, pycnidiospores. Scale bar = 10 μm.

The effect of ulvan on the *in planta* epiphytic growth of *Z. tritici* was investigated by staining the fungus with Calcofluor. At 1 dai, the percentage of germinated spores over the leaf surface of water-treated plants was 59%. No significant effect of ulvan treatment on the *in planta* spore germination was observed when compared with the control (Figures 3C, 4A,B). At 5 dai, the percentage of non-germinated spores (NGS), germinated spores with a short germ tube (GS-SGT), germinated spores with a well-developed germ tube (GS-DGT), and germinated spores with branched hyphae (GS-BH) were 14.1, 22.5, 27.1, and 37.7% in water-treated plants, respectively. Likewise, ulvan spraying did not significantly impact the fungal epiphytic development *in planta* when compared to the control (Figures 3D, 4C,D).

### Ulvan Elicits but Does Not Prime the Expression of Targeted Defense Genes

**FIGURE 5:**
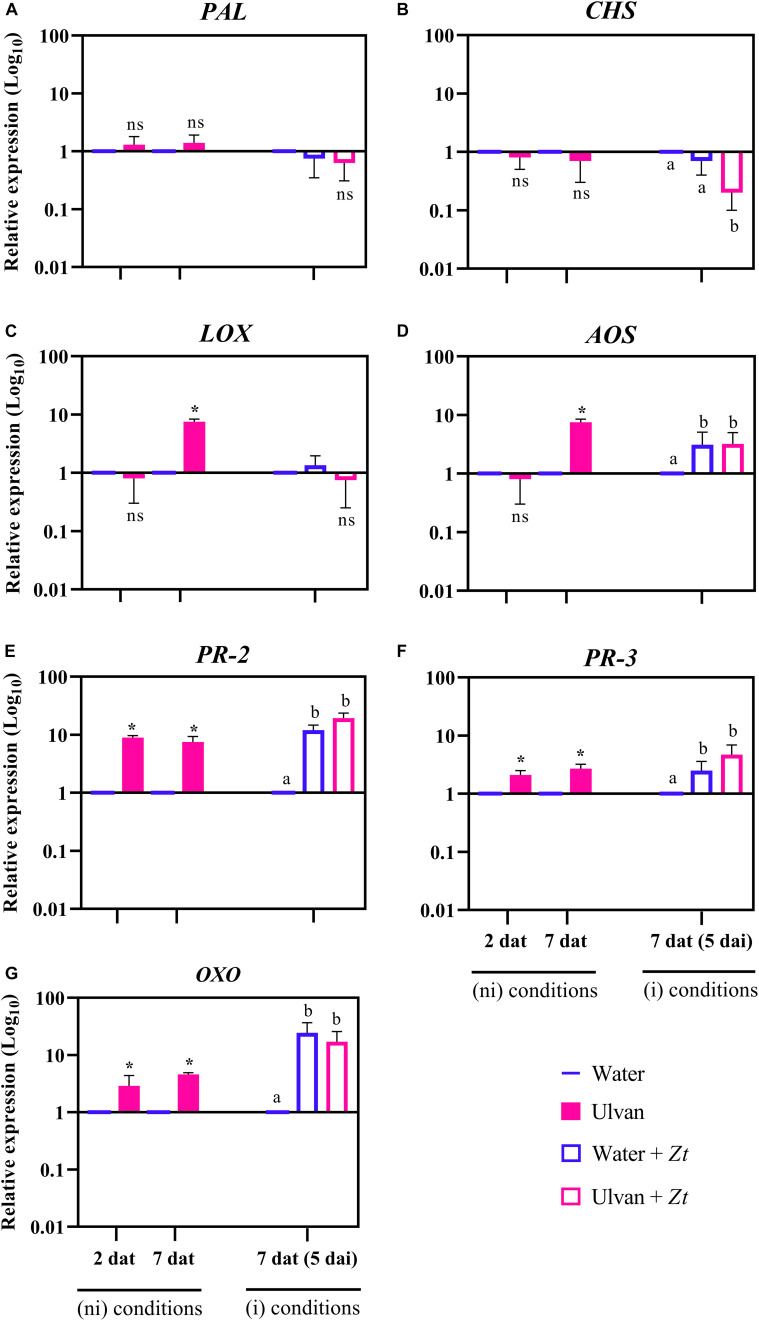
Relative expression of phenylalanine ammonia-lyase (*PAL*; **A**), chalcone synthase (*CHS*; **B**), lipoxygenase (*LOX*; **C**), allene oxide synthase (*AOS*; **D**), β-1,3-endoglucanase (*PR-2*; **E**), chitinase 2 (*PR-3*; **F**), and oxalate oxidase (*OXO*; **G**) in wheat plants (cv. Alixan) at 2 and 7 days after treatment (dat) with ulvan, with 7 dat corresponding to 5 days after inoculation (dai) with *Zymoseptoria tritici* (*Zt*). The relative expression was calculated as Log10 fold change compared to water-sprayed non-inoculated (control) plants at each time point. The expression value of the control was fixed at 1. Asterisks and letters indicate significant differences according to the Wilcoxon–Mann–Whitney and Kruskal–Wallis tests at *P* ≤ 0.05, respectively. ns, not significant when compared to the control. Plants were inoculated 2 days after treatment. ni, non-inoculated; i, inoculated.

The relative expression of the seven genes involved in wheat defense reactions—namely, phenylalanine ammonia-lyase (*PAL*), chalcone synthase (*CHS*), lipoxygenase (*LOX*), allene oxide synthase (*AOS*), β-1,3-endoglucanase (*PR-2*), chitinase 2 (*PR-3*), and oxalate oxidase (*OXO*)—were analyzed using RT-qPCR in water- (control) and ulvan-treated plants prior to and after infection with *Z. tritici*. In non-inoculated conditions, at 2 dat, ulvan increased the expression of *PR-2*, *PR-3*, and *OXO* by 8.9-, 2.3-, and 2.9-fold compared with the control, respectively (Figures 5E–G). The expression of these three genes remained higher in ulvan-treated non-inoculated plants at 7 dat, i.e., 7.5-, 2.7-, and 4.6-fold for *PR-2*, *PR-3*, and *OXO*, respectively. In addition, an upregulation of *LOX* and *AOS* with 7.5-fold was also observed in ulvan-treated non-inoculated plants at 7 dat (Figures 5C,D). When compared to non-inoculated plants in absence of ulvan pretreatment, the inoculation with *Z. tritici* induced an upregulation of *AOS*, *PR-2*, *PR-3*, and *OXO* by 3.0-, 12.0-, 2.5-, and 24.4-fold at 5 dai, respectively (Figures 5D–G). On the other hand, pretreatment with ulvan repressed the expression of *CHS* by 0.3-fold in infected plants at 5 dai (Figure 5B). No significant modulation of *PAL*, *PR-2*, *PR-3*, *AOS*, *LOX*, and *OXO* was observed in ulvan-treated inoculated plants compared to inoculated control ones (Figures 5A,C–G).

### Ulvan Did Not Alter Wheat Metabolome in Both Non-infectious and Infectious Contexts

**FIGURE 6:**
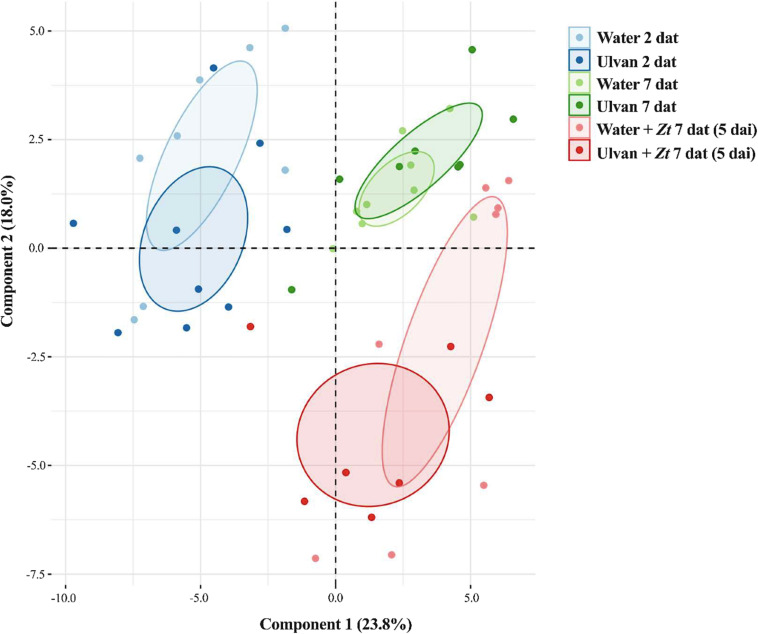
Principal component analysis (PCA) of global wheat leaf metabolite levels at 2 and 7 days after treatment (dat) with ulvan, with 7 dat corresponding to 5 days after inoculation (dai) with *Zymoseptoria tritici* (*Zt*). Plants were inoculated 2 days after treatment.

To quantify relative amounts of a total of 83 compounds in wheat leaves sprayed or not with ulvan, UHPLC-MS was performed (Supplementary Figure 1 and Supplementary Table 3). The selected metabolites were grouped into 10 chemical families, i.e., amines, amino acids, benzoxazinoids, carboxylic acids, coumarins, flavonoids, hormones, hydroxycinnamic acid amides, sugars, and terpenoids. The PCA analysis of metabolite amounts resulted in different groups according to sampling time after ulvan treatment, i.e., 2 and 7 dat and between non-inoculated and inoculated plants at 5 dai, where the first and second component explained 23.8% and 18% of the variation, respectively, totalizing 41.8% of variations (Figure 6).

**FIGURE 7:**
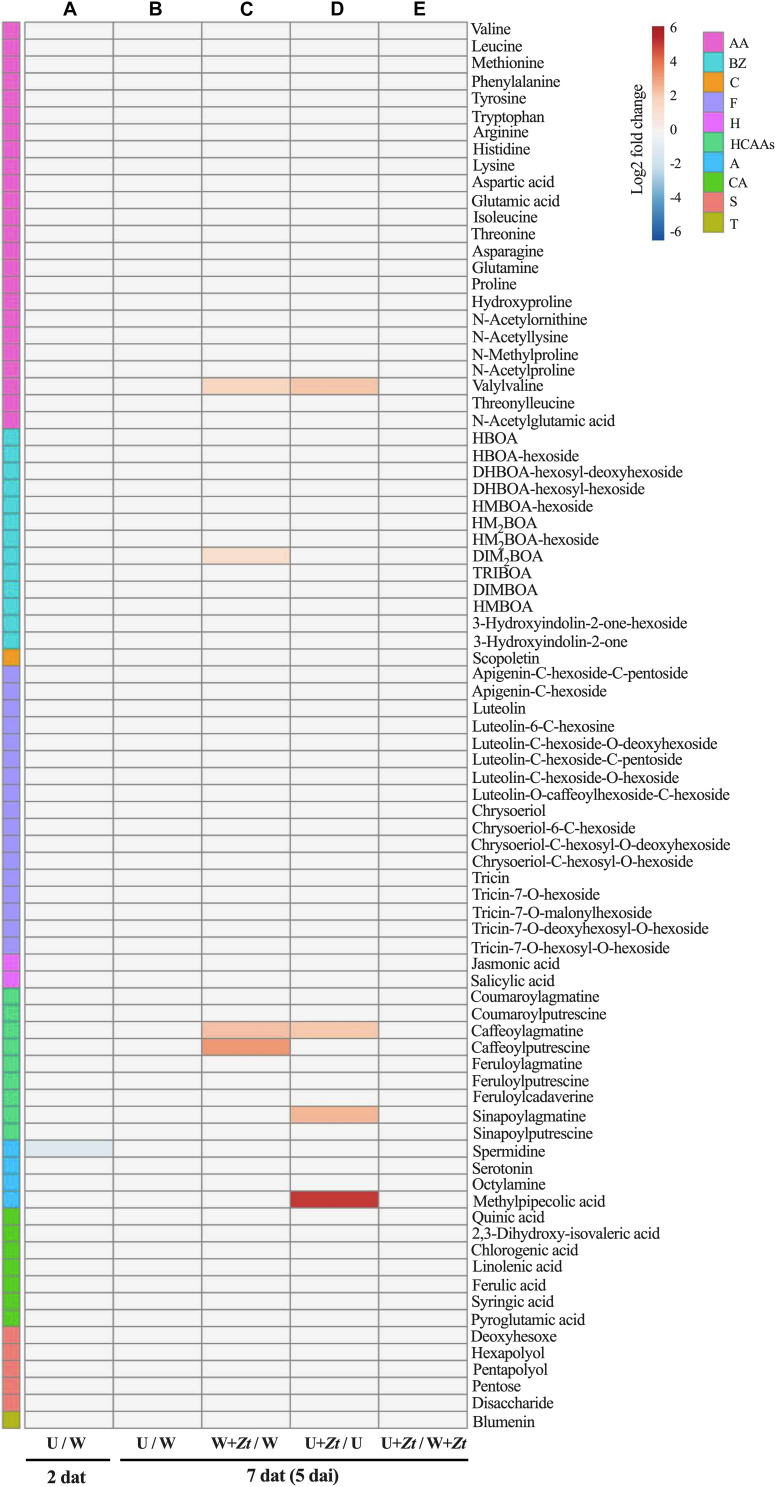
Heatmap of metabolite fold changes in wheat leaves at 2 **(A)** and 7 **(B–E)** days after treatment (dat) with ulvan, with 7 dat corresponding to 5 days after inoculation (dai) with *Zymoseptoria tritici* (*Zt*). Upregulated and downregulated metabolite Log2 fold changes are indicated by red and blue colors, respectively. Metabolites were grouped according to their chemical family as amines (A), amino acids (AA), benzoxazinoids (BZ), carboxylic acids (CA), coumarins (C), flavonoids (F), hormones (H), hydroxycinnamic acid amides (HCAAs), sugars (S), and terpenoids (T). Pairwise comparisons were performed using Tukey’s honest significant difference method followed by a false discovery rate (FDR) correction, with FDR < 0.05. For FDR ≥ 0.05, Log2 fold changes were set to 0. W, water; U, ulvan; W + *Zt*, Water + inoculation with *Zt*; U + *Zt*, Ulvan + inoculation with *Zt*.

Differential analysis was then performed to identify the potential impact of different treatments on the selected metabolites (Figure 7). When comparing treatments in non-inoculated conditions, ulvan did not significantly change the levels of these metabolites, with the exception of spermidine that was significantly downregulated (≤0.55-fold) at 2 dat (Figures 7A,B and Supplementary Table 4). When compared to non-inoculated plants, the infection of *Z. tritici* upregulated the levels of six metabolites at 5 dai (≥3.3-fold); namely, methylpipecolic acid (amines), valylvaline (amino acids), DIM_2_BOA (benzoxazinoids), caffeoylagmatine, caffeoylputrescine, and sinapoylagmatine (hydroxycinnamic acid amides) (Figures 7C,D and Supplementary Table 4). On the other hand, pretreatment with ulvan did not significantly change the levels of the studied metabolites in inoculated plants at 5 dai when compared with water-treated inoculated controls (Figure 7E and Supplementary Table 4).

## Discussion

Research to develop eco-friendly plant protection compounds without negative impacts on the environment and human health and fitting with the concept of sustainable agriculture is strongly encouraged nowadays. In this study, we report for the first time the potential of ulvan, a sulfated heteropolysaccharide isolated from the green macroalgae *Ulva fasciata*, for conferring significant protection to wheat against *Z. tritici*, a major fungal pathogen in this crop. This finding agrees with previous studies that demonstrated the ability of ulvan to enhance plant resistance against phytopathogens (Stadnik and de Freitas, 2014).

Our results showed that ulvan did not affect neither spore germination nor mycelial or hyphal growth of *Z. tritici* in both *in vitro* and *in planta* assays, thus corroborating previous works where no direct antifungal effect of ulvan toward distinct pathogens including fungi, yeast, and bacteria has been reported (Paulert et al., 2007, 2009; de Freitas and Stadnik, 2012). In contrast, we found that ulvan spraying significantly reduced the colonization of substomatal cavities in wheat leaves; more specifically, the rate of substomatal cavities bearing pycnidia, indicating that the protection conferred by ulvan treatment is associated with the specific defense responses of the plant rather than direct antifungal effects on the pathogen. Consequently, we analyzed the monosaccharide composition of ulvan and performed dedicated gene expression and metabolomic assays to explore the molecular basis of ulvan-induced resistance in the wheat-*Z. tritici* pathosystem.

The MALDI-TOF-MS analysis indicated that ulvan is mainly composed of unsaturated monosaccharides (rhamnose, rhamnose-3-sulfate, and xylose) and numerous uronic acid residues. Rhamnose is a major monosaccharide component of ulvan (Paulert et al., 2009; de Freitas et al., 2015) and is also present in rhamnogalacturonans (Pettolino et al., 2012) and rhamnolipids (Varnier et al., 2009), while uronic acids participate in the composition of polysaccharides in most life forms (Pettolino et al., 2012). Molecules containing rhamnose or uronic acids are known to trigger plant defense responses against pathogens (Varnier et al., 2009; de Freitas et al., 2015). As evidenced by Jaulneau et al. (2010), our work reinforces the proposition that ulvan recognition by the plant may be associated with rhamnose and/or uronic acid residues. However, the signaling mechanisms underlying their perception remain to be further determined.

Molecular investigations revealed that ulvan induced several plant defense-related genes in non-inoculated wheat plants, including genes encoding for PR proteins (*PR-2* and *PR-3*), ROS metabolism (*OXO*), and the octadecanoid-based pathway (*LOX* and *AOS*). Interestingly, some of these genes such as *PR-2*, *PR-3*, and *OXO* were upregulated at both 2 and 7 dat, suggesting the eliciting effect of ulvan on wheat plants. The ability of ulvan to elicit the expression of plant defense genes has been previously observed in *Medicago truncatula* (Cluzet et al., 2004). In wheat, the application of extracts from another seaweed, such as the *Ascophyllum nodosum*-based product Dalgin Active^®^, has been reported to elicit *PR-2* and *LOX* (Somai-Jemmali et al., 2020). As a consequence of this enhanced basal resistance, plants may have more success in defending themselves against further fungal attacks. Indeed, it has been suggested that an upregulation of *PR-2* and *PR-3* genes, encoding for glucanase and chitinase enzymes, respectively, could reduce *Z. tritici* colonization by their known role in digesting β-glucans and chitin of the fungal cell wall, respectively (Shetty et al., 2009; Ors et al., 2019; Somai-Jemmali et al., 2020). In our case, we did not observe a significant upregulation of these targeted genes upon ulvan treatment in infected conditions. Therefore, taken together, our results indicate that the defense mechanisms triggered by ulvan in wheat against *Z. tritici* are more likely associated with an eliciting effect rather than with a priming effect. Accordingly, the eliciting effect of ulvan on glucanase activity was also reported in non-inoculated bean plants, even in those resistant to the hemibiotrophic fungus *Colletotrichum lindemuthianum* (de Freitas and Stadnik, 2012). On the other hand, using a system of suspension-cultured wheat cells, Paulert et al. (2010) concluded that ulvan have the priming effect because they did not elicit the production of hydrogen peroxide alone. Indeed, ulvan-pretreated wheat cells exhibited a stronger chitin-elicited oxidative burst response than the non-pretreated cells. Hence, looking at the earlier times of fungal infection to elucidate if ulvan also has a priming effect on wheat plants will be an exciting challenge for future research.

Wheat infection by *Z. tritici* upregulated the expression of *AOS*, *PR-2*, *PR-3*, and *OXO* genes at 5 dai independently of ulvan pre-treatment. This response may be associated with the basal defense reactions triggered by the plant in an attempt to defend itself against the pathogen, as evidenced by Ors et al. (2018). However, we cannot exclude the possibility that the fungus has invested efforts in activating specific plant defense responses such as *OXO* to support further colonization and disease development as well. This is because *OXO* is responsible for the conversion of oxalic acid and oxygen (O_2_) into carbon dioxide (CO_2_) and hydrogen peroxide (H_2_O_2_). Although H_2_O_2_ can be harmful to *Z. tritici* throughout its life cycle, this fungus can tolerate high levels of H_2_O_2_ (Shetty et al., 2007) and use it to induce host cell collapse mainly at the transition to the necrotrophic phase and reproduction (Keon et al., 2007). Alternatively, the high expression of *OXO* observed in our study may be the consequence of stress-related responses due to fungal infection rather than an efficient defense mechanism. Indeed, large-scale activation of the ROS metabolism has been observed after infection of *Z. tritici* in susceptible cultivars (Keon et al., 2007; Rudd et al., 2015; Ors et al., 2018).

Pre-treatment of ulvan downregulated the expression of *CHS* at 5 dai, and no modulation of *PAL* was observed at this time point. This result indicates that the biosynthesis of phenylpropanoids is probably not involved in the induction of plant resistance conferred by ulvan in wheat. Accordingly, no significant upregulation of flavonoids or coumarins was observed in our metabolomic analysis. The activation of *PAL* and *CHS* genes has been involved with the basal resistance of wheat (Adhikari et al., 2007; Ors et al., 2018) and after induction by the application of inducers, but mainly in resistant genotypes (Ors et al., 2019). Similarly, no induction of *PAL* was observed after treatment with the Dalgin Active^®^, inducer in susceptible plants (Somai-Jemmali et al., 2020), suggesting that the activation of the phenylpropanoid pathway in bread wheat may be a cultivar- and/or elicitor-dependent response.

Targeted UHPLC-MS analyses were carried out to quantify the relative amounts of the 83 leaf metabolites belonging to major chemical families. Consistent with previous findings, infection by *Z. tritici* resulted in the accumulation of significant amounts of hydroxycinnamic acid amides (HCAAs) defense compounds (Seybold et al., 2020). Interestingly, the accumulation of a derivative of pipecolic acid, identified as methylpipecolic acid, was highly stimulated upon infection by *Z. tritici*. Lysine metabolism to pipecolic acid derivatives has been shown to be an important activator of defense reactions in several plant species (Hartmann and Zeier, 2018). In contrast, these metabolomic analyses did not reveal substantial changes in the leaf metabolome following ulvan treatment in both non-infected and infected conditions. Indeed, ulvan spraying did not significantly change the levels of the studied metabolites, with the exception of a slight downregulation of spermidine at 2 dat. As the analyzed metabolites were extracted with methanol, we cannot exclude that ulvan treatment may impact methanol-insoluble compounds, such as polymers involved in the reinforcement of cell walls. Altogether, our results suggest that ulvan treatment does not induce major changes in a selection of important leaf metabolites belonging to various chemical families. This observation reinforces the interest in ulvan as a plant health promoter, as the ulvan-conferred protection in wheat does not seem to be associated with important metabolic costs.

The induction of defense responses might be energetically costly for the plant (Walters and Heil, 2007). Such expenses are normally associated with increased demands for nitrogen and carbon skeletons that are provided by the primary metabolic pathways, such as carbohydrates and amino acids (Bolton, 2009). Our metabolic findings are in agreement with Cluzet et al. (2004), who highlighted that ulvan treatment-induced resistance in *Medicago truncatula* against *Colletotrichum trifolii* infection did not alter plant primary metabolism.

In summary, our study reports for the first time the potential of ulvan to protect wheat against *Z. tritici*. The displayed protection efficacy relies on plant resistance induction since ulvan did not exbibit any direct antifungal effect toward the pathogen in both *in vitro* and *in planta* conditions. Gene expression analysis indicated that ulvan induces resistance in wheat against *Z. tritici via* an eliciting effect rather than a priming effect and that this eliciting effect in wheat relies on the induction of PR protein synthesis, ROS metabolism, and octadecanoids, but do not on the phenylpropanoids. Interestingly, there was no evidence for major alteration in the wheat leaf metabolome upon ulvan treatment during the biotrophic phase of fungal infection, suggesting low plant metabolic and fitness costs associated with ulvan-induced resistance.

## Data Availability Statement

The original contributions presented in the study are included in the article/Supplementary Material, further inquiries can be directed to the corresponding authors.

## Author Contributions

MB performed all experiments and wrote the first draft of the manuscript. CF performed the MALDI-TOF-MS analysis. MB, MH, MM-R, BR, and AV performed the gene expression assays and related data analyses. MB, AM-G, RB, and PH performed the metabolomic assays and related data analysis. J-LH contributed to the whole data analyses and the manuscript writing. PR, MS, and AS supervised this study. All authors contributed to the article and approved the submitted version.

## Conflict of Interest

The authors declare that the research was conducted in the absence of any commercial or financial relationships that could be construed as a potential conflict of interest.

## Publisher’s Note

All claims expressed in this article are solely those of the authors and do not necessarily represent those of their affiliated organizations, or those of the publisher, the editors and the reviewers. Any product that may be evaluated in this article, or claim that may be made by its manufacturer, is not guaranteed or endorsed by the publisher.

## Footnotes

1
http://www.genome.ad.jp/kegg/

2
http://pubchem.ncbi.nlm.nih.gov

## Supplementary Material

Supplementary Figure 1 Heatmap of global wheat leaf metabolite levels at 2 and 7 days after treatment (dat) with ulvan, with 7 dat corresponding to 5 days after inoculation (dai) with *Zymoseptoria tritici* (*Zt*). Log10 mean peak area of the indicated metabolites is given by shades of red or blue colors according to the scale bar. Metabolites were grouped according to their chemical family as amines (A), amino acids (AA), benzoxazinoids (BZ), carboxylic acids (CA), coumarins (C), flavonoids (F), hormones (H), hydroxycinnamic acid amides (Hcaas), sugars (S), and terpenoids (T). W, water; U, ulvan; W + *Zt*, Water + inoculation with *Zt*; U + *Zt*, Ulvan + inoculation with *Zt*.

Supplementary Table 1 Primer sequences, GenBank accession numbers and corresponding references for genes studied in the quantitative reverse-transcription polymerase chain reaction (RT-qPCR) assays.

Supplementary Table 2 Monosaccharide composition of ulvan and related average theoretical molecular mass.

Supplementary Table 3 List of the 83 metabolites detected in wheat leaves (cv. Alixan) with ultra-high-performance liquid chromatography-mass spectrometry (UHPLC-MS), using a suspect screening strategy – targeted metabolomic approach.

Supplementary Table 4 Changes in metabolite levels in wheat leaves at 2 and 7 days after treatment (dat) with ulvan, with 7 dat corresponding to 5 days after inoculation (dai) with *Zymoseptoria tritici*.
