# Supplementary material for: The Algal Polysaccharide Ulvan Induces Resistance in Wheat Against Zymoseptoria tritici Without Major Alteration of Leaf Metabolome
Source: Front Plant Sci. 2021 Sep 6;12:703712. doi: 10.3389/fpls.2021.703712 (PMC8450535; doi:10.3389/fpls.2021.703712)
Supplement: Supplementary Figure 1 — Heatmap of global wheat leaf metabolite levels at 2 and 7 days after treatment (dat) with ulvan, with 7 dat corresponding to 5 days after inoculation (dai) with Zymoseptoria tritici (Zt). Log10 mean peak area of the indicated metabolites is given by shades of red or blue colors according to the scale bar. Metabolites were grouped according to their chemical family as amines (A), amino acids (AA), benzoxazinoids (BZ), carboxylic acids (CA), coumarins (C), flavonoids (F), hormones (H), hydroxycinnamic acid amides (Hcaas), sugars (S), and terpenoids (T). W, water; U, ulvan; W + Zt, Water + inoculation with Zt; U + Zt, Ulvan + inoculation with Zt. [file Data_Sheet_1.docx]

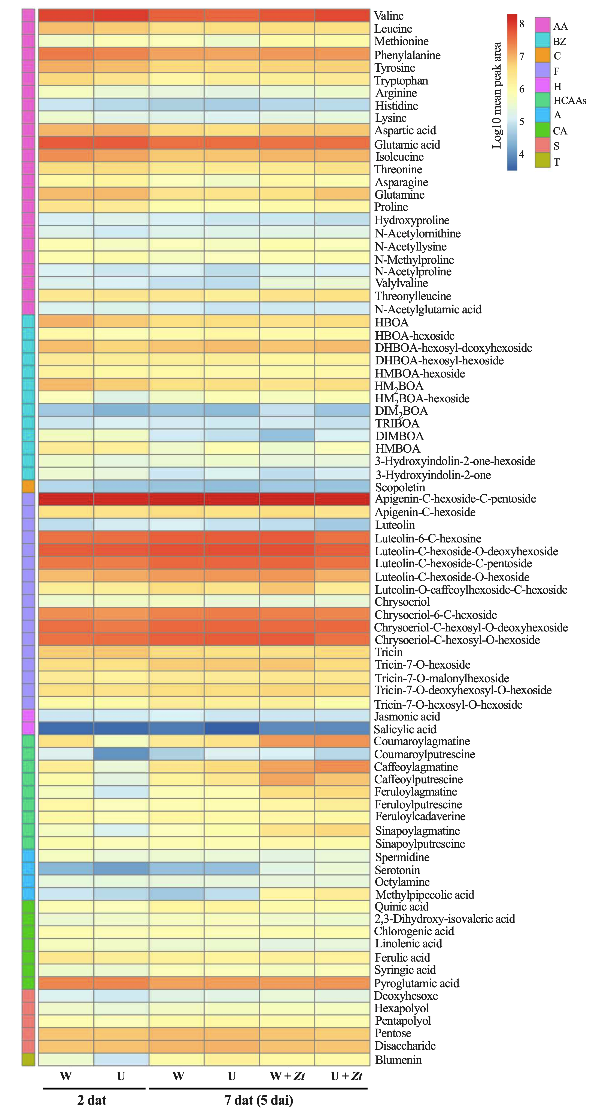


**Supplementary Figure 1 |** Heatmap of global wheat leaf metabolite levels at 2 and 7 days after treatment (dat) with ulvan, with 7 dat corresponding to 5 days after inoculation (dai) with Zymoseptoria tritici (Zt). Log10 mean peak area of the indicated metabolites is given by shades of red or blue colors according to the scale bar. Metabolites were grouped according to their chemical family as amines (A), amino acids (AA), benzoxazinoids (BZ), carboxylic acids (CA), coumarins (C), flavonoids (F), hormones (H), hydroxycinnamic acid amides (Hcaas), sugars (S), and terpenoids (T). W, water; U, ulvan; W + Zt, Water + inoculation with Zt; U + Zt, Ulvan + inoculation with Zt.

**TABLE S1 |** Primer sequences, GenBank accession numbers and corresponding references for genes studied in the quantitative reverse-transcription polymerase chain reaction (RT-qPCR) assays.

| **Gene** | **Primer sequence (5′-3′)** | **GenBank accession number** | **References** |
| --- | --- | --- | --- |
| *TUB* (β-tubulin 4, Housekeeping Gene) | GGAGTACCCTGACCGAATGATG  AACGACGGTGTCTGAGACCTTT | U76895 | Tayeh et al., 2013 |
| *PetA* (Class A Apetala 2, Housekeeping Gene) | CCTGCCCCGTACAACCTTGAG  CACCGTTGCGATAGTCCTGAAAC | AB749309 | This study |
| *PAL* (Phenylalanine Ammonia lyase) | CCCCCATTGGTGTCTCCAT  ACTGCGCGAACATCAGCTT | AY005474 | Tayeh et al., 2015 |
| *CHS* (Chalcone Synthase) | GCGCCTGCGTACTCTTCATC  CCTCGGCGGAGCGTTT | AY286097 | Ors et al., 2018 |
| *LOX* (Lipoxygenase) | GGGCACCAAGGAGTACAAGGA  GCTCGTGATGGTGTGGATGA | U32428 | Tayeh et al., 2015 |
| *AOS* (Allene Oxide Synthase) | AGGCCGGAGAGAAGTTCCAC  CCGACTTGGTCAGCTCCATC | AY196004 | Ors et al., 2018 |
| *PR-2* (β-1,3-endoglucanase) | TCCTGGGTTCAGAACAATGTCC  TTGATGTTGACAGCCGGGTAGT | DQ090946 | Ors et al., 2018 |
| *PR-3* (Chitinase 2) | GGGTGGACCTGCTGAACAAT  AGAACCATATCGCCGTCTTGA | AB029935 | Ors et al., 2018 |
| *OXO* (Oxalate Oxidase) | GCCAGAACCCCGGTATCG  GGTGGGTTGGAGCTGAAGAG | AJ556991 | Ors et al., 2018 |

**TABLE S2 |** Monosaccharide composition of ulvan and related average theoretical molecular mass.

| **Monosaccharide composition** | **Detailed calculation of average theoretical molecular mass** | **Average theoretical molecular mass [M]** | **Average theoretical *m/z* ([M-H]^-^)** |  |
| --- | --- | --- | --- | --- |
| (∆) / Rha3S / Xyl | (-18.01) + 244.21 + (-18.01) + 150.13 | 358.32 | 357.32 |  |
| Rha / Xyl / Rha | 164.16 + (-18.01) + 150.13 + (-18.01) + 164.16 | 442.43 | 441.43 |  |
| (∆)_2_ / Rha3S / Xyl / Rha | (-18.01)*2 + 244.22 + (-18.01) + 150.13 + (-18.01) + 164.16 | 486.47 | 485.47 |  |
| (∆) / Rha3S / Xyl / Rha | (-18.01) + 244.22 + (-18.01) + 150.13 + (-18.01) + 164.16 | 504.48 | 503.48 |  |
| Rha / Xyl / Rha / HexA | 164.16 + (-18.01) + 150.13 + (-18.01) + 164.16 + (-18.01) + 194.14 | 618.56 | 617.56 |  |
| (∆)_2_ / Rha3S / Xyl / Rha / HexA | (-18.01)*2 + 244.22 + (-18.01) + 150.13 + (-18.01) + 164.16 + (-18.01) + 194.14 | 662.60 | 661.60 |  |
| (∆) / Rha3S / Xyl / Rha / HexA | (-18.01) + 244.22 + (-18.01) + 150.13 + (-18.01) + 164.16 + (-18.01) + 194.14 | 680.61 | 679.61 |  |
| (∆) / Rha3S / Xyl / Rha / (HexA)_2_ | (-18.01) + 244.22 + (-18.01) + 150.13 + (-18.01) + 164.16 + ((-18.01) + 194.14)*2 | 856.74 | 855.74 |  |
| (∆) / Rha3S / Xyl / Rha / (HexA)_3_ | (-18.01) + 244.22 + (-18.01) + 150.13 + (-18.01) + 164.16 + ((-18.01) + 194.14)*3 | 1032.87 | 1031.87 |  |
| (∆) / Rha3S / Xyl / Rha / (HexA)_4_ | (-18.01) + 244.22 + (-18.01) + 150.13 + (-18.01) + 164.16 + ((-18.01) + 194.14)*4 | 1209.00 | 1208.00 |  |
| (∆) / Rha3S / Xyl / Rha / (HexA)_5_ | (-18.01) + 244.22 + (-18.01) + 150.13 + (-18.01) + 164.16 + ((-18.01) + 194.14)*5 | 1385.13 | 1384.13 |  |
| (∆) / Rha3S / Xyl / Rha / (HexA)_6_ | (-18.01) + 244.22 + (-18.01) + 150.13 + (-18.01) + 164.16 + ((-18.01) + 194.14)*6 | 1561.26 | 1560.26 |  |
| (∆) / Rha3S / Xyl / Rha / (HexA)_7_ | (-18.01) + 244.22 + (-18.01) + 150.13 + (-18.01) + 164.16 + ((-18.01) + 194.14)*7 | 1737.39 | 1736.39 |  |
| Unsaturation (∆) can be located either on Rha3S, Rha or HexA; rhamnose-3-sulfate (Rha3S); rhamnose (Rha); xylose (Xyl); uronic acid (HexA). Unsaturation and osidic linkages correspond to -18.01 atomic mass unit. | | | | |

**TABLE S3** **|** List of the 83 metabolites detected in wheat leaves (cv. Alixan) with ultra-high-performance liquid chromatography-mass spectrometry (UHPLC-MS), using a suspect screening strategy – targeted metabolomic approach.

| **Id** | **Metabolite** | **Formula** | **Family ^a^** | **Detected *m/z*** | ***m/z* error (ppm)** | **RT (min)** | **KEGG ID** | **PubChem CID** |
| --- | --- | --- | --- | --- | --- | --- | --- | --- |
| 1 | Methylpipecolic acid | C_7_H_13_NO_2_ | Amines | 144.1019 | 0.27 | 1.32 |  | 415939 |
| 2 | Octylamine | C_8_H_19_N | Amines | 130.1591 | 0.71 | 4.52 | [C01740](https://www.genome.jp/dbget-bin/www_bget?cpd:C01740) | [8143](https://pubchem.ncbi.nlm.nih.gov/compound/Octylamine) |
| 3 | Serotonin | C_10_H_12_N_2_O | Amines | 177.1023 | 0.23 | 4.44 | [C00780](https://www.genome.jp/dbget-bin/www_bget?C00780) | [5202](https://pubchem.ncbi.nlm.nih.gov/compound/serotonin) |
| 4 | Spermidine | C_7_H_19_N_3_ | Amines | 146.1653 | 0.54 | 0.85 | [C00315](https://www.genome.jp/dbget-bin/www_bget?cpd:C00315) | [1102](https://pubchem.ncbi.nlm.nih.gov/compound/spermidine) |
| 5 | Arginine* | C_6_H_14_N_4_O_2_ | Amino acids | 175.1190 | 0.20 | 0.95 | [C00062](https://www.genome.jp/dbget-bin/www_bget?C00062) | [6322](https://pubchem.ncbi.nlm.nih.gov/compound/L-arginine) |
| 6 | Asparagine* | C_4_H_8_N_2_O_3_ | Amino acids | 133.0609 | 0.86 | 1.17 | [C00152](https://www.genome.jp/dbget-bin/www_bget?C00152) | [6267](https://pubchem.ncbi.nlm.nih.gov/compound/L-asparagine) |
| 7 | Aspartic acid* | C_4_H_7_NO_4_ | Amino acids | 134.0449 | 0.69 | 1.09 | [C00049](https://www.genome.jp/dbget-bin/www_bget?C00049) | [5960](https://pubchem.ncbi.nlm.nih.gov/compound/L-aspartic_acid) |
| 8 | Glutamic acid* | C_5_H_9_NO_4_ | Amino acids | 148.0604 | -0.58 | 1.17 | [C00025](https://www.genome.jp/dbget-bin/www_bget?C00025) | [4525487](https://pubchem.ncbi.nlm.nih.gov/compound/4525487) |
| 9 | Glutamine* | C_5_H_10_N_2_O_3_ | Amino acids | 147.0764 | 0.09 | 1.08 | [C00064](https://www.genome.jp/dbget-bin/www_bget?C00064) | [5961](https://pubchem.ncbi.nlm.nih.gov/compound/L-glutamine) |
| 10 | Histidine* | C_6_H_9_N_3_O_2_ | Amino acids | 156.0769 | 0.68 | 1.00 | [C00135](https://www.genome.jp/dbget-bin/www_bget?C00135) | [6274](https://pubchem.ncbi.nlm.nih.gov/compound/6274) |
| 11 | Hydroxyproline* | C_5_H_9_NO_3_ | Amino acids | 132.0656 | 0.70 | 1.27 | [C01157](https://www.genome.jp/dbget-bin/www_bget?C01157) | [5810](https://pubchem.ncbi.nlm.nih.gov/compound/5810) |
| 12 | Isoleucine* | C_6_H_13_NO_2_ | Amino acids | 132.1020 | 0.76 | 2.96 | [C00407](https://www.genome.jp/dbget-bin/www_bget?C00407) | [6306](https://pubchem.ncbi.nlm.nih.gov/compound/l-isoleucine) |
| 13 | Leucine* | C_6_H_13_NO_2_ | Amino acids | 132.1020 | 0.76 | 2.76 | [C00123](https://www.genome.jp/dbget-bin/www_bget?C00123) | [6106](https://pubchem.ncbi.nlm.nih.gov/compound/6106) |
| 14 | Lysine* | C_6_H_14_N_2_O_2_ | Amino acids | 147.1129 | 0.44 | 0.84 | [C00047](https://www.genome.jp/dbget-bin/www_bget?cpd:C00047) | 5962 |
| 15 | Methionine* | C_5_H_11_NO_2_S | Amino acids | 150.0584 | 0.36 | 2.04 | [C00073](https://www.genome.jp/dbget-bin/www_bget?C00073) | [6137](https://pubchem.ncbi.nlm.nih.gov/compound/L-methionine) |
| 16 | N-Methylproline | C_6_H_11_NO_2_ | Amino acids | 130.0863 | 0.37 | 1.31 |  | [643474](https://pubchem.ncbi.nlm.nih.gov/compound/N-Methyl-L-proline) |
| 17 | N-Acetyllysine | C_8_H_16_N_2_O_3_ | Amino acids | 189.1234 | 0.15 | 4.50 | [C02727](https://www.genome.jp/dbget-bin/www_bget?cpd+C02727) | [92832](https://pubchem.ncbi.nlm.nih.gov/compound/N-epsilon-Acetyl-L-lysine) |
| 18 | N-Acetylglutamic acid | C_7_H_11_NO_5_ | Amino acids | 190.0711 | 0.56 | 1.12 | [C00624](https://www.genome.jp/dbget-bin/www_bget?cpd:C00624) | [70914](https://pubchem.ncbi.nlm.nih.gov/compound/70914) |
| 19 | N-Acetylornithine | C_7_H_14_N_2_O_3_ | Amino acids | 175.1077 | 0.13 | 1.34 | [C00437](https://www.genome.jp/dbget-bin/www_bget?C00437) | [439232](https://pubchem.ncbi.nlm.nih.gov/compound/N-acetylornithine) |
| 20 | N-Acetylproline | C_7_H_11_NO_3_ | Amino acids | 158.0812 | 0.24 | 1.34 |  | [66141](https://pubchem.ncbi.nlm.nih.gov/compound/N-Acetyl-L-proline) |
| 21 | Phenylalanine* | C_9_H_11_NO_2_ | Amino acids | 166.0862 | -0.53 | 4.30 | [C00079](https://www.genome.jp/dbget-bin/www_bget?C00079) | [6140](https://pubchem.ncbi.nlm.nih.gov/compound/6140) |
| 22 | Proline* | C_5_H_9_NO_2_ | Amino acids | 116.0708 | 1.69 | 1.23 | [C00148](https://www.genome.jp/dbget-bin/www_bget?C00148) | [145742](https://pubchem.ncbi.nlm.nih.gov/compound/L-proline) |
| 23 | Threonine* | C_4_H_9_NO_3_ | Amino acids | 120.0657 | 1.22 | 1.08 | [C00188](https://www.genome.jp/dbget-bin/www_bget?C00188) | [6288](https://pubchem.ncbi.nlm.nih.gov/compound/6288) |
| 24 | Threonylleucine | C_10_H_20_N_2_O_4_ | Amino acids | 233.1495 | -0.20 | 4.29 |  | [7021828](https://pubchem.ncbi.nlm.nih.gov/compound/H-Thr-Leu-OH) |
| 25 | Tryptophan* | C_11_H_12_N_2_O_2_ | Amino acids | 205.0973 | 0.51 | 4.75 | [C00078](https://www.genome.jp/dbget-bin/www_bget?C00078) | [6305](https://pubchem.ncbi.nlm.nih.gov/compound/6305) |
| 26 | Tyrosine* | C_9_H_11_NO_3_ | Amino acids | 182.0813 | 0.54 | 3.20 | [C00082](https://www.genome.jp/dbget-bin/www_bget?C00082) | [6057](https://pubchem.ncbi.nlm.nih.gov/compound/6057) |
| 27 | Valine* | C_5_H_11_NO_2_ | Amino acids | 118.0863 | 0.48 | 1.14 | [C00183](https://www.genome.jp/dbget-bin/www_bget?cpd:C00183) | [6287](https://pubchem.ncbi.nlm.nih.gov/compound/L-valine) |
| 28 | Valylvaline | C_10_H_20_N_2_O_3_ | Amino acids | 217.1547 | -0.03 | 4.27 |  | [409682](https://pubchem.ncbi.nlm.nih.gov/compound/Valylvaline) |
| 29 | 3-Hydroxyindolin-2-one | C_8_H_7_NO_2_ | Benzoxazinoids | 150.0550 | 0.15 | 5.14 | [C11130](https://www.genome.jp/dbget-bin/www_bget?C11130) | [6097](https://pubchem.ncbi.nlm.nih.gov/compound/Dioxindole) |
| 30 | 3-Hydroxyindolin-2-one-hexoside | C_14_H_17_NO_7_ | Benzoxazinoids | 312.1077 | 0.02 | 4.75 |  |  |
| 31 | DHBOA-hexosyl-hexoside | C_14_H_17_NO_9_ | Benzoxazinoids | 344.2300 | 0.59 | 4.48 |  |  |
| 32 | DHBOA-hexosyl-deoxyhexoside | C_20_H_27_NO_14_ | Benzoxazinoids | 506.3400 | 0.59 | 4.71 |  |  |
| 33 | DIMBOA | C_9_H_9_NO_5_ | Benzoxazinoids | 212.0555 | 0.62 | 5.40 | [C04720](https://www.genome.jp/dbget-bin/www_bget?cpd:C04720) | [2358](https://pubchem.ncbi.nlm.nih.gov/compound/dimboa) |
| 34 | DIM_2_BOA | C_10_H_11_NO_6_ | Benzoxazinoids | 242.0660 | 0.14 | 4.31 |  | [11139254](https://pubchem.ncbi.nlm.nih.gov/compound/11139254) |
| 35 | HBOA | C_8_H_7_NO_3_ | Benzoxazinoids | 166.0499 | 0.34 | 5.92 | [C15769](https://www.genome.jp/dbget-bin/www_bget?cpd:C15769) | [322636](https://pubchem.ncbi.nlm.nih.gov/compound/hboa) |
| 36 | HBOA-hexoside | C_14_H_17_NO_8_ | Benzoxazinoids | 328.1027 | 0.09 | 4.71 |  | [14605136](https://pubchem.ncbi.nlm.nih.gov/compound/14605136) |
| 37 | HM_2_BOA | C_10_H_11_NO_5_ | Benzoxazinoids | 226.0709 | -0.61 | 5.51 |  |  |
| 38 | HMBOA-hexoside | C_15_H_19_NO_9_ | Benzoxazinoids | 358.1241 | -0.61 | 5.06 |  |  |
| 39 | HM_2_BOA-hexoside | C_16_H_21_NO_10_ | Benzoxazinoids | 388.1245 | 1.86 | 5.36 |  |  |
| 40 | HMBOA | C_9_H_9_NO_4_ | Benzoxazinoids | 196.0605 | 0.42 | 5.42 |  | [152213](https://pubchem.ncbi.nlm.nih.gov/compound/HMBOA) |
| 41 | TRIBOA | C_8_H_7_NO_5_ | Benzoxazinoids | 198.0398 | 0.64 | 4.20 |  | [10965465](https://pubchem.ncbi.nlm.nih.gov/compound/10965465) |
| 42 | Scopoletin | C_10_H_8_O_4_ | Coumarins | 193.0496 | 0.53 | 5.65 | [C01752](https://www.genome.jp/dbget-bin/www_bget?C01752) | [5280460](https://pubchem.ncbi.nlm.nih.gov/compound/scopoletin) |
| 43 | 2,3-Dihydroxy-isovalerate | C_5_H_10_O_4_ | Carboxylic acids | 135.0653 | 0.57 | 1.30 | [C04039](https://www.genome.jp/dbget-bin/www_bget?C04039) | [440279](https://pubchem.ncbi.nlm.nih.gov/compound/440279) |
| 44 | Chlorogenic acid* | C_16_H_18_O_9_ | Carboxylic acids | 355.1024 | -0.01 | 5.21 | [C00852](https://www.genome.jp/dbget-bin/www_bget?C00852) | [1794427](https://pubchem.ncbi.nlm.nih.gov/compound/chlorogenic_acid) |
| 45 | Ferulic acid* | C_10_H_10_O_4_ | Carboxylic acids | 195.0652 | 0.24 | 5.05 | [C01494](https://www.genome.jp/dbget-bin/www_bget?cpd:C01494) | [445858](https://pubchem.ncbi.nlm.nih.gov/compound/ferulic_acid) |
| 46 | Linolenic acid* | C_18_H_30_O_2_ | Carboxylic acids | 279.2319 | 0.12 | 9.36 | C06427 | 5280934 |
| 47 | Pyroglutamic acid | C_5_H_7_NO_3_ | Carboxylic acids | 130.0526 | 0.11 | 1.17 | C01879 | 7405 |
| 48 | Quinic acid* | C_7_H_12_O_6_ | Carboxylic acids | 193.0707 | -0.08 | 1.16 | [C00296](https://www.genome.jp/dbget-bin/www_bget?C00296) | [6508](https://pubchem.ncbi.nlm.nih.gov/compound/quinic_acid) |
| 49 | Syringic acid | C_9_H_10_O_5_ | Carboxylic acids | 199.0602 | 0.56 | 4.65 | [C10833](https://www.genome.jp/dbget-bin/www_bget?C10833) | [10742](https://pubchem.ncbi.nlm.nih.gov/compound/syringic_acid) |
| 50 | Apigenin-6-C-hexoside | C_21_H_20_O_10_ | Flavonoids | 433.1129 | -0.16 | 5.18 | [C01714](https://www.genome.jp/dbget-bin/www_bget?C01714) | [162350](https://pubchem.ncbi.nlm.nih.gov/compound/Isovitexin) |
| 51 | Apigenin-C-hexoside-C-pentoside | C_26_H_28_O_14_ | Flavonoids | 565.1553 | 0.27 | 5.05 |  |  |
| 52 | Chrysoeriol | C_16_H_12_O_6_ | Flavonoids | 301.0707 | 0.05 | 6.42 | [C04293](https://www.genome.jp/dbget-bin/www_bget?C04293) | [5280666](https://pubchem.ncbi.nlm.nih.gov/compound/Chrysoeriol) |
| 53 | Chrysoeriol-6-C-hexoside | C_22_H_22_O_11_ | Flavonoids | 463.1237 | 0.43 | 5.35 | [C05990](https://www.genome.jp/dbget-bin/www_bget?cpd:C05990) | [442611](https://pubchem.ncbi.nlm.nih.gov/compound/Isoscoparin) |
| 54 | Chrysoeriol-C-hexosyl-O-deoxyhexoside | C_28_H_32_O_15_ | Flavonoids | 609.1817 | 0.50 | 5.24 |  |  |
| 55 | Chrysoeriol-C-hexosyl-O-hexoside | C_28_H_32_O_16_ | Flavonoids | 625.1766 | 0.42 | 5.17 |  |  |
| 56 | Luteolin | C_15_H_10_O_6_ | Flavonoids | 287.0549 | -0.40 | 6.05 | [C01514](https://www.genome.jp/dbget-bin/www_bget?C01514) | [5280445](https://pubchem.ncbi.nlm.nih.gov/compound/luteolin) |
| 57 | Luteolin-6-C-hexoside | C_21_H_20_O_11_ | Flavonoids | 449.1080 | 0.30 | 5.00 |  |  |
| 58 | Luteolin-C-hexoside-O-hexoside | C_27_H_30_O_16_ | Flavonoids | 611.1607 | 0.50 | 5.01 |  |  |
| 59 | Luteolin-C-hexoside-O-pentoside | C_26_H_28_O_15_ | Flavonoids | 581.1504 | 0.51 | 4.97 |  |  |
| 60 | Luteolin-C-hexoside-O-deoxyhexoside | C_27_H_30_O_15_ | Flavonoids | 595.1660 | 0.45 | 5.02 |  |  |
| 61 | Luteolin-O-caffeoylhexoside-C-hexoside | C_36_H_36_O_19_ | Flavonoids | 773.1928 | 0.51 | 5.07 |  |  |
| 62 | Tricin | C_17_H_14_O_7_ | Flavonoids | 331.0812 | -0.07 | 6.45 | [C10193](https://www.genome.jp/dbget-bin/www_bget?cpd:C10193) | [5281702](https://pubchem.ncbi.nlm.nih.gov/compound/Tricin) |
| 63 | Tricin-7-O-hexoside | C_23_H_24_O_12_ | Flavonoids | 493.1343 | 0.58 | 5.56 |  | [5322022](https://pubchem.ncbi.nlm.nih.gov/compound/5322022) |
| 64 | Tricin 7-O-malonylhexoside | C_26_H_26_O_15_ | Flavonoids | 579.1348 | 0.55 | 5.68 |  | [122391240](https://pubchem.ncbi.nlm.nih.gov/compound/122391240) |
| 65 | Tricin 7-O-deoxyhexosyl-O-hexoside | C_29_H_34_O_16_ | Flavonoids | 639.1924 | 0.66 | 5.27 |  |  |
| 66 | Tricin 7-O-hexosyl-O-hexoside | C_29_H_34_O_17_ | Flavonoids | 655.1869 | 0.02 | 5.28 |  |  |
| 67 | Jasmonic acid* | C_12_H_18_O_3_ | Hormones | 211.1329 | 0.23 | 6.27 | [C08491](https://www.genome.jp/dbget-bin/www_bget?C08491) | [5281166](https://pubchem.ncbi.nlm.nih.gov/compound/Jasmonic_acid) |
| 68 | Salicylic acid* | C_7_H_6_O_3_ | Hormones | 139.0390 | 0.44 | 6.35 | [C00805](https://www.genome.jp/dbget-bin/www_bget?C00805) | [338](https://pubchem.ncbi.nlm.nih.gov/compound/salicylic_acid) |
| 69 | Caffeoylagmatine | C_14_H_20_N_4_O_3_ | Hydroxycinnamic acid amides | 293.1608 | 0.06 | 4.52 |  | 129662439 |
| 70 | Caffeoylputrescine | C_13_H_18_N_2_O_3_ | Hydroxycinnamic acid amides | 251.1390 | 0.08 | 4.47 | C03002 | 5280559 |
| 71 | Coumaroylagmatine | [C_14_H_20_N_4_O_2_](https://pubchem.ncbi.nlm.nih.gov/#query=C14H20N4O2) | Hydroxycinnamic acid amides | 277.1659 | 0.01 | 4.66 | C04498 | [5280691](https://pubchem.ncbi.nlm.nih.gov/compound/5280691) |
| 72 | Coumaroylputrescine | C_13_H_18_N_2_O_2_ | Hydroxycinnamic acid amides | 235.1441 | 0.02 | 4.50 | C18326 | 6439562 |
| 73 | Feruloylagmatine | C_15_H_22_N_4_O_3_ | Hydroxycinnamic acid amides | 307.1765 | -0.05 | 4.57 | [C18325](https://www.genome.jp/dbget-bin/www_bget?C18325) | [46173376](https://pubchem.ncbi.nlm.nih.gov/compound/46173376) |
| 74 | Feruloylcadaverine | [C_15_H_22_N_2_O_3_](https://pubchem.ncbi.nlm.nih.gov/#query=C15H22N2O3) | Hydroxycinnamic acid amides | 279.1702 | 0.43 | 4.85 |  | 44420470 |
| 75 | Feruloylputrescine | C_14_H_20_N_2_O_3_ | Hydroxycinnamic acid amides | 265.1548 | 0.32 | 4.70 | [C10497](https://www.genome.jp/dbget-bin/www_bget?C10497) | [5281796](https://pubchem.ncbi.nlm.nih.gov/compound/Feruloylputrescine) |
| 76 | Sinapoylagmatine | C_16_H_24_N_4_O_4_ | Hydroxycinnamic acid amides | 337.1870 | 0.09 | 4.65 |  | 129662367 |
| 77 | Sinapoylputrescine | C_15_H_22_N_2_O_4_ | Hydroxycinnamic acid amides | 295.1653 | 0.22 | 4.56 |  | 131751429 |
| 78 | Deoxyhexose | C_6_H_12_O_5_ | Sugars | 165.0757 | 0.3 | 1.05 |  |  |
| 79 | Disaccharide | C_12_H_22_O_11_ | Sugars | 343.1234 | -0.13 | 1.36 |  |  |
| 80 | Pentose | C_5_H_10_O_5_ | Sugars | 151.0601 | 0.03 | 2.13 |  |  |
| 81 | Hexapolyol | C_6_H_14_O_6_ | Sugar alcohols | 183.0863 | -0.06 | 1.17 |  |  |
| 82 | Pentapolyol | C_5_H_12_O_5_ | Sugar alcohols | 153.0758 | 0.06 | 1.3 |  |  |
| 83 | Blumenin | C_25_H_40_O_13_ | Terpenoids | 549.2545 | 0.64 | 5.07 |  | [101708178](https://pubchem.ncbi.nlm.nih.gov/compound/101708178) |
| ^a^ Family was classified according to KEGG (Kyoto Encyclopedia of Genes and Genomes; <http://www.genome.ad.jp/kegg>) and PubChem (<http://pubchem.ncbi.nlm.nih.gov>) databases. Id: identifier; *m/z*: mass-to-charge ratio; *m/z* error (difference between the measured *m/z* and the calculated *m/z* of an ion, represented in ppm); RT: retention time. * indicates that compounds were confirmed with authentic standards. | | | | | | | | |

**TABLE S4 |** Changes in metabolite levels in wheat leaves at 2 and 7 days after treatment (dat) with ulvan, with 7 dat corresponding to 5 days after inoculation (dai) with *Zymoseptoria tritici*.

| **Id** | **Metabolite** | **Formula** | **Family ^a^** | **Detected *m/z*** | **RT (min)** | **Fold change (compared to the control)** | | | | |  |
| --- | --- | --- | --- | --- | --- | --- | --- | --- | --- | --- | --- |
|  |  |  |  |  |  | **2 dat** |  | **7 dat (5 dai)** | | |  |
|  |  |  |  |  |  | **U/W ^b^** |  | **U/W ^b^** | **W+*Zt*/W ^b^** | **U+*Zt*/U ^c^** | **U+*Zt*/W+*Zt* ^d^** |
| 1 | Spermidine | C_7_H_19_N_3_ | Amines | 146.1653 | 0.85 | 0.55** |  | - | - | - | - |
| 2 | Methylpipecolic acid | C_7_H_13_NO_2_ | Amines | 144.1019 | 1.32 | - |  | - | - | 32.7** | - |
| 3 | Valylvaline | C_10_H_20_N_2_O_3_ | Amino acids | 217.1547 | 4.27 | - |  | - | 3.4** | 4.2** | - |
| 4 | DIM_2_BOA | C_10_H_11_NO_6_ | Benzoxazinoids | 242.0660 | 4.31 | - |  | - | 3.3* | - | - |
| 5 | Caffeoylagmatine | C_14_H_20_N_4_O_3_ | Hydroxycinnamic acid amides | 293.1608 | 4.52 | - |  | - | 5.9** | 3.8* | - |
| 6 | Caffeoylputrescine | C_13_H_18_N_2_O_3_ | Hydroxycinnamic acid amides | 251.1390 | 4.47 | - |  | - | 16.6** | - | - |
| 7 | Sinapoylagmatine | C_16_H_24_N_4_O_4_ | Hydroxycinnamic acid amides | 337.1870 | 4.65 | - |  | - | - | 5.44** | - |
| ^a^ Family was classified according to KEGG (<http://www.genome.ad.jp/kegg/>) and PubChem (<http://pubchem.ncbi.nlm.nih.gov>) databases. ^b^ Plants sprayed with water + Tween 20 served as controls. ^c^ Plants sprayed with ulvan + Tween 20 served as controls. ^d^ Plants inoculated with *Zymoseptoria tritici* (*Zt*) + Tween 20 were used as controls. Id: identifier; *m/z*: mass-to-charge ratio; RT: retention time; W, Water; U, Ulvan; W + *Zt*, Water + inoculation with *Zt*; U + *Zt*, Ulvan + inoculation with *Zt*; -, not significantly changed compared to the control. * and ** indicates significant difference compared to the control according to the Student´s t-test at *P* ≤ 0.01 and *P* ≤ 0.001, respectively. | | | | | | | | | | | |
